# The “Histoplasmosis Porto Alegre manifesto”—Addressing disseminated histoplasmosis in AIDS

**DOI:** 10.1371/journal.pntd.0010960

**Published:** 2023-01-05

**Authors:** Alessandro C. Pasqualotto, Flavio Queiroz-Telles, Alberto Chebabo, Terezinha M. J. S. Leitao, Diego R. Falci, Melissa O. Xavier, Monica B. Bay, Eduardo Sprinz, Daiane Dalla Lana, Adriana P. Vincentini, Lisandra Serra Damasceno, Alexandre V. Schwarzbold, Paulo Abrão Ferreira, Cassia Miranda Godoy, Jose Ernesto Vidal, Rossana Basso, Candida Driemeyer, Valerio R. Aquino, Cecilia B. Severo, Marcelo Simão Ferreira, Claudilson Bastos, Filipe Prohaska, Marineide Melo, Francelise Bridi Cavassin, Marcus Lacerda, Renata Soares, Rosely Zancope-Oliveira, Marcus Teixeira, Freddy Perez, Diego H. Caceres, Juan Luis Rodriguez-Tudela, Tom Chiller, Arnaldo L. Colombo

**Affiliations:** 1 Universidade Federal de Ciências da Saúde, Porto Alegre, Brazil; 2 Universidade Federal do Paraná, Curitiba, Brazil; 3 Universidade Federal do Rio de Janeiro, Rio de Janeiro, Brazil; 4 Universidade Federal do Ceará, Fortaleza, Brazil; 5 Pontifícia Universidade Católica do Rio Grande do Sul, Porto Alegre, Brazil; 6 Universidade Federal de Rio Grande, Rio Grande, Brazil; 7 Universidade Federal do Rio Grande do Norte, Natal, Brazil; 8 Universidade Federal do Rio Grande do Sul, Porto Alegre, Brazil; 9 Instituto Adolfo Lutz, São Paulo, Brazil; 10 Hospital São José, Fortaleza, Brazil; 11 Universidade Federal de Santa Maria, Santa Maria, Brazil; 12 Universidade Federal de São Paulo, São Paulo, Brazil; 13 Pontifícia Universidade Católica de Goiás, Goiânia, Brazil; 14 Instituto Emilio Ribas, São Paulo, Brazil; 15 Hospital de Clínicas de Porto Alegre, Porto Alegre, Brazil; 16 Universidade Federal de Uberlândia, Uberlândia, Brazil; 17 Universidade Estadual do Sudoeste da Bahia, Salvador, Brazil; 18 Faculdade de Medicina de Olinda, Ouro Preto, Brazil; 19 Grupo Hospitalar Conceição, Porto Alegre, Brazil; 20 Fundação de Medicina Tropical Dr. Heitor Vieira Dourado, Manaus, Brazil; 21 Fundação Oswaldo Cruz, Rio de Janeiro, Brazil; 22 Universidade de Brasília, Brasília, Brazil; 23 Panamerican Health Organization, Washington, DC, United States of America; 24 IMMY, Norman, Oklahoma, United States of America; 25 Instituto de Salud Carlos III, Madrid, Spain; 26 Center for Disease Control and Prevention, Atlanta, Georgia, United States of America; Centre Hospitalier Universitaire de Besancon, FRANCE

Histoplasmosis is a global fungal disease that has a high prevalence especially in the Americas caused by diverse species nested within the genus *Histoplasma* [1]. Other parts of the world where it has been identified include Southern Europe, Africa, and Asia [2]. In Latin America, histoplasmosis is the most prevalent systemic endemic mycoses [3]. The disease is associated with a wide plethora of clinical manifestations, including disseminated histoplasmosis (DH) in immunocompromised patients, chronic pulmonary disease, pulmonary nodules, mediastinal adenitis, fibrosing mediastinitis, chorioretinitis, and acute pulmonary disease. For many of these conditions, the differential diagnosis with tuberculosis is challenging. Risk populations for DH include people living with HIV/AIDS (PLWHA) and other immunocompromising conditions, like transplant recipients, and extremes of age and the use of immunosuppressive medicines, like the use of steroids and TNF-α inhibitors [4]. Reported cases certainly underestimate the actual burden of histoplasmosis, since in most places, *Histoplasma* antigen detection was not available, and many states did not report the occurrence of cases.

Etiological diagnosis of infectious diseases has a central role in medicine. Clinicians need a clear diagnosis so they can initiate appropriate treatment for the best care of their patients. The laboratory is key for the diagnosis of infectious diseases. However, for life-threatening infections, it is essential to give therapies as quickly as possible, to decrease mortality. Microscopy is the traditional method that can offer a rapid response for DH, but its sensitivity is low and very dependent on the observer expertise, the clinical sample, and fungal burden. For instance, microscopy of oral lesions may allow for a quick diagnosis, but this already represents late (disseminated) disease. These limitations make it a challenging technique, and its reported high sensitivity is based on publications made by experts that do not take into account the reality of variability in the training and capacities of health workers. Histopathology needs highly trained professionals, and misdiagnoses are frequent. Furthermore, tissue biopsies for specific fungal stains are not always easy to obtain. Although histopathology can be quicker than culture, several days are still needed to get a reliable result. Culture of clinical samples is considered the gold standard, but it is positive in only 50% to 85% of cases (most often from bone marrow aspirate, skin, or lymph node biopsies), and it does not provide a rapid result since the fungus can take up to 4 to 6 weeks to grow. So relying on culture alone often means that the disease is treated empirically and based on the clinician’s experience with histoplasmosis, which, in many settings, is far from adequate (not to mention that many people are erroneously treated empirically for tuberculosis). A prospective study involving 4,245 patients (of which 227 had histoplasmosis) showed that culture of respiratory samples for recovering *Histoplasma* spp. was only positive in only 8.5% (95% CI, 5.4% to 13.0%) and Isolator blood culture in 36.3% (95% CI, 29.8% to 43.3%) [5], which means the need to perform invasive procedures like bone marrow sampling to increase sensitivity. In addition, culture requires Biosafety Level 3 containment, which is not available in many settings, putting health workers managing the culture samples in the filamentous form of the fungus at risk of acquiring the disease. Finally, although antibody detection could be applied to the presumptive diagnosis of histoplasmosis, it is not commercially available. Furthermore, it has a low sensitivity in immunocompromised patients (highest sensitivity is seen with western blot technique)—not to mention the lack of standardization in *Histoplasma* antigen preparations [6,7]. Antibody detection may be of special interest for the immunocompetent patient, including travelers from nonendemic areas.

The landscape of histoplasmosis diagnosis has changed since the introduction of antigen detection tests. They are performed in urine, blood, and serum—relatively easy and inexpensive samples to obtain—and the results can be obtained on the same day, with some formats in a couple of hours. Urine is usually preferred, and the test can be done with TB LAM, since disseminated tuberculosis is a common condition in advanced AIDS. The training is simple and not highly technical. The performance of antigen tests is superior to all diagnostic tests with a sensitivity and specificity over 90%. As they are commercially available, a proficiency quality control program is easy to set up to validate the quality of the laboratory results. These tests have proven to diagnose more cases when compared to conventional methods as well as having an impact on patients’ survival [8–10]. The performance of *Histoplasma* antigen testing in the non-HIV population deserves further investigation.

Other tools will still be needed to diagnose localized histoplasmosis. Several in-house real-time PCR-based approaches have proven that this technique is a valuable addition to diagnosis portfolio. A recent study has shown that a combination of tests should be employed, with PCR the most common after antigen detection, in order to detect all types of histoplasmosis in PLWHA [5]. qPCR assays are on the road for commercial availability and can also detect *Histoplasma* DNA in patients with disseminated disease, using blood samples [11]. Of note, previous studies were based on nested PCR [5], which may not perform as well as modern qPCR assays for the diagnosis of histoplasmosis. The availability of a commercial histoplasmosis real-time PCR diagnosis system would be welcomed by the fungal disease global community.

The real incidence of histoplasmosis remains unclear since antigen detection is far from universally available and the disease is generally not reportable to public health authorities. However, several surveys conducted in sentinel centers of endemic areas have consistently documented high morbidity and mortality rates. For instance, a modelling study using prevalence of histoplasmin-positive reactions and the incidence of HIV in Latin America led to an estimate of 1.1 to 6.7K annual deaths that could be attributed to histoplasmosis [12]. A survey done with histoplasmin intradermal tests in HIV–positive patients with CD4 counts >350 cells/mm^3^, living in an hyperendemic area in the Northeast Brazil, showed a prevalence of positive reactions of 11.8% [13], demonstrating the frequency of fungus exposure in a high-risk population for severe forms of this illness. Centers from the Northeast region of Brazil (42.3%) and from the Central-West region (53.0%) reported very high fatality rates for DH, emphasizing the impact of this condition in the country [7]. Recent data using antigen detection testing [8] have shown that histoplasmosis is a very common condition in PLWHA admitted to the hospital, with prevalence rates over 40% in the Northeast and Central-West regions. This study also showed a trend to less mortality when a positive antigen detection test precedes a diagnosis by conventional methods (14.3% versus 26.9%), which corroborates the observation that in North America, where antigen detection is widely available, mortality rates of DH are significantly lower (around 10%) [8,14]. In Guatemala, a similar trend towards reduced mortality in PLWHA when *Histoplasma* antigen was made available was observed, which was probably linked to an earlier and quicker diagnosis [10].

Considering the high incidence, morbidity, and mortality of histoplasmosis in PLWHA in Brazil, there is a need for public health authorities to take further steps to combat these diseases. In particular, diagnostic tools allowing for early and effective treatment need to be put in place in all locations where high-risk patients are seen, that should apply to other underlying conditions leading to DH. Furthermore, patients need to be treated with the best formulation of amphotericin B—liposomal amphotericin B (L-AmB)—instead of the more toxic compound amphotericin B deoxycholate (d-AmB).

The accumulated evidence and experience is clear that the use of *Histoplasma* antigen detection has the potential to reduce mortality due to histoplasmosis in high-risk patients. Providing a rapid result allows clinicians to make treatment decisions days and even weeks before [and with higher sensitivity than] culture or skin lesions appear and consequently increasing the chances of survival. Therefore, there was a clear consensus during the *Brazilian Histoplasmosis Meeting* (Porto Alegre, May 2022) that access to *Histoplasma* antigen testing is critical to reduce the mortality of histoplasmosis as well as to generate accurate data on the real burden of histoplasmosis in different geographic regions and risk populations. Such diagnostic tools should be made widely available, both for public and private hospitals.

In terms of critical antifungal treatment, L-AmB use is supported by clinical trials and international guidelines as being the first choice for DH, being superior to d-AmB in both efficacy and toxicity [15,16]. It will be critical to determine how to guarantee universal access to L-AmB in the different regions of Brazil. Of course, access to itraconazole is also required for long-term treatment, but this drug is more frequently available in Latin American countries.

Once we have full access to *Histoplasma* urinary antigen and L-AmB for both diagnosis and treatment of DH, patients will get the life-saving treatment they need in a timely manner. It is also important to better understand the natural history of histoplasmosis by implementing universal antigen screening in patients at high risk, which could allow for preemptive treatment that may prevent the development of a disseminated disease in these high-risk patients. A number of other strategies that need to be investigated include the following: (i) short (high-dose regimens) induction courses of L-AmB decreasing the length of hospitalization, side effects, and costs (as observed for cryptococcosis) [17]; (ii) the use of *Histoplasma* antigen clearance or *Histoplasma* DNA to monitor antifungal therapy, therefore individualizing duration of therapy; (iii) determining the frequency of histoplasmosis and tuberculosis codetection, and how to best treat these individuals; (iv) reducing duration of antifungal therapy in PLWA responding to antiretroviral therapy; (v) efficacy of preemptive antifungal therapy in patients with positive *Histoplasma* antigen or qPCR; (vi) investigation of novel therapies or artificial intelligence drug repositioning that would be effective in patients with multiple comorbidities and histoplasmosis; and (vii) strategies to mitigate negative impact of antifungal interactions (for instance, itraconazole) in patients under antiretroviral therapy with histoplasmosis.

In conclusion, to decrease the morbidity and mortality of DH, the panel of experts stresses that two critical and urgent factors are required in the Brazilian health system: capacity to diagnose with rapid antigen test detection, and access to L-AmB (Box 1). These claims are in consonance with previous work from GAFFI and the histoplasmosis advocacy group, recently published as the “Manaus declaration” [18].

**Box 1. Main needs related to histoplasmosis in Brazil**

- Histoplasmosis is endemic in Brazil; therefore, proper disease awareness is needed, particularly in high-risk patients such as those with advanced HIV disease.
- Early diagnosis of disseminated histoplasmosis (DH) requires access to *Histoplasma* antigen detection.
- All patients with DH should have access to liposomal amphotericin B.
