# Correction: The “Histoplasmosis Porto Alegre manifesto”—Addressing disseminated histoplasmosis in AIDS

**DOI:** 10.1371/journal.pntd.0011106

**Published:** 2023-01-31

**Authors:** The PLOS Neglected Tropical Diseases Staff

The tenth author’s name is spelled incorrectly. The correct name is: Adriana P. Vicentini.

## References

[1] Pasqualotto AC, Queiroz-Telles F, Chebabo A, Leitao TMJS, Falci DR, Xavier MO, et al. (2023) The “Histoplasmosis Porto Alegre manifesto”—Addressing disseminated histoplasmosis in AIDS. PLoS Negl Trop Dis 17(1): e0010960. 10.1371/journal.pntd.0010960 36602963 PMC9815569

